# High yield recombinant penicillin G amidase production and export into the growth medium using *Bacillus megaterium*

**DOI:** 10.1186/1475-2859-5-36

**Published:** 2006-11-28

**Authors:** Yang Yang, Rebekka Biedendieck, Wei Wang, Martin Gamer, Marco Malten, Dieter Jahn, Wolf-Dieter Deckwer

**Affiliations:** 1 Biochemical Engineering, TU-BCE, HZI-Helmholtz Centre for Infection Research, Inhoffenstrasse 7, 38124 Braunschweig, Germany; 2 Institute of Microbiology, Technical University Braunschweig, Spielmannstraße 7, D-38106 Braunschweig, Germany

## Abstract

**Background:**

During the last years *B. megaterium *was continuously developed as production host for the secretion of proteins into the growth medium. Here, recombinant production and export of *B. megaterium *ATCC14945 penicillin G amidase (PGA) which is used in the reverse synthesis of β-lactam antibiotics were systematically improved.

**Results:**

For this purpose, the PGA leader peptide was replaced by the *B. megaterium *LipA counterpart. A production strain deficient in the extracellular protease NprM and in xylose utilization to prevent gene inducer deprivation was constructed and employed. A buffered mineral medium containing calcium ions and defined amino acid supplements for optimal PGA production was developed in microscale cultivations and scaled up to a 2 Liter bioreactor. Productivities of up to 40 mg PGA per L growth medium were reached.

**Conclusion:**

The combination of genetic and medium optimization led to an overall 7-fold improvement of PGA production and export in *B. megaterium*. The exclusion of certain amino acids from the minimal medium led for the first time to higher volumetric PGA activities than obtained for complex medium cultivations.

## Background

The Gram positive bacterium *Bacillus megaterium *has several advantages over other microbial host-systems for the production and secretion of recombinant proteins [1]. In contrast to *Escherichia coli *it has a high capacity for protein export [2]. Compared to *Bacillus subtilis*, *B. megaterium *reveals an useful plasmid stability and a low intrinsic protease activity [2]. Important prerequisites for a biotechnological application of this organism include an efficient transformation system, multiple compatible, freely replicating plasmids and the possibility to integrate a heterologous gene into the genome [3,4]. Recently, production of heterologous exoproteins by *B. megaterium *was further improved by use the exoprotease NprM-deficient *B. megaterium *strain MS941 [5,6] and by the coexpression of the signal peptidase gene *sipM *[7]. However, some bottlenecks were still observed for the production and secretion of some of the studied heterologous proteins. The multidomain and high molecular weight dextransucrase DsrS (M_r _= 180,000) from *Leuconostoc mesenteroides *aggregated extracellularly during high cell density cultivation [1]. The heterologous gene of the *Thermobifida fusca *hydrolase (*tfh*) was only successfully expressed in *B. megaterium *after its codon bias was adapted to *B. megaterium *codon usage [8]. Other unknown limiting factors contained in the employed semi-defined medium repressed protein production and secretion in high cell density cultivation [8].

Here, we report on the expression of the penicillin G amidase gene (*pga*) isolated from *B. megaterium *ATCC14945 in derivatives of *B. megaterium *DSM319. This homologous penicillin G amidase (PGA) has a relative molecular mass of 90,000 consisting of two autocatalytically processed subunits (α, β) [9]. The function of PGA in nature is not yet fully understood. *B. megaterium *may produce PGA extracellularly to degrade phenylacetylated compounds in order to generate phenylacetic acid (PAA) which can be used as carbon source [10]. In industry, PGA is used for the production of new β-lactam antibiotics. It hydrolyzes penicillin G yielding phenyl acetate and 6-aminopenicillanic acid (6-APA). The 6-APA provides the molecular core of all β-lactams to which D-amino acid derivatives can be substituted to create novel antibiotics, *e.g*. amoxicillin. PGA of *B. megaterium *is industrially used for the outlined reverse synthesis reaction due to its higher synthesis rate compared to *E. coli *PGA [11,12]. The intensively studied *E. coli *PGA is predominantly exported into the periplasm [10]. In contrast, using *B. megaterium *to secrete homologous *B. megaterium *PGA directly into the growth medium should facilitate its purification and consequently decrease the downstream processing and final production costs.

In this contribution we tested directed molecular strategies for the stepwise improvement of PGA production and secretion using *B. megaterium*.

## Results

### Rationale of the experimental approach for PGA production in *B. megaterium*

First, in order to stabilize the desired product PGA in the growth medium the influence of calcium ions and the extracellular protease NprM on enzyme stability and activity were investigated. Subsequently, the leader peptide of the extracellular lipase LipA from *B. megaterium *was tested for the improvement of PGA export. Gene induction using the *xylA *promoter was analyzed in a *xylA *mutant strain to prevent inducer utilization. Finally, medium optimization and up scaling were approached systematically.

### Increased recombinant PGA production and secretion using *B. megaterium *by the addition of calcium ions

**Figure 1:**
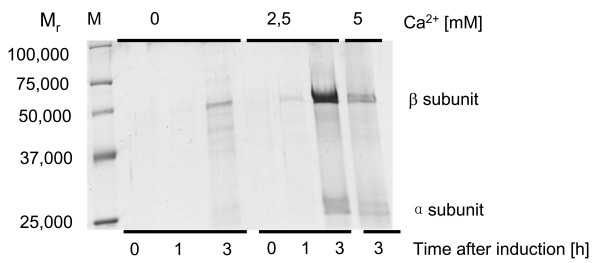
Influence of calcium ions on PGA production and export in *B. megaterium*. MS941 carrying pRBBm23 (encoding SP_*pga*_-PGA) was cultivated in LB medium with indicated concentrations of CaCl_2_. Proteins from 1.5 mL cell-free growth medium were precipitated by ammonium sulfate, analyzed by SDS-PAGE and stained with Coomassie Brilliant Blue G250. Lane M shows Precision Plus Protein Standard (Bio-Rad, Muenchen, Germany).

Previous investigations of homologous PGA production in *E. coli *identified calcium as an important factor for protein folding and maturation [13,14]. An amino acid sequence alignment of PGA from *B. megaterium *and *E. coli *showed that all active site amino acids were conserved. An overall amino acid sequence identity of 28.4 % was observed. Although the degree of sequence identity is low, functionally and structurally important amino acids were found conserved, indicating homology at the structural level. Hence, the influence of calcium ions on the activity of *B. megaterium *PGA was tested. The complete *pga *gene was cloned into the *Bsr*GI/*Sac*I site of pMM1522 placing its expression under control of the xylose inducible promoter. The new vector pRBBm23 was transformed into protoplasted *B. megaterium *MS941 cells. This *B. megaterium *strain is deficient in the major extracellular protease NprM due to deletion of the corresponding gene. Significant stabilization of exported proteins by *B. megaterium *MS941 was reported before [5,7]. The influence of different calcium ion concentrations on the secretion of recombinant PGA was tested in shaking flask cultivations. Comparing the addition of various calcium ion concentrations to the complex LB growth medium demonstrated that 2.5 mM CaCl_2 _was optimal for PGA production (Fig. 1). Three hours after induction 189.4, 489.9, and 287.3 U PGA g_CDW _^-1 ^were measured in the growth medium containing none, 2.5, and 5 mM CaCl_2_, respectively. The addition of 2.5 mM CaCl_2 _increased the amount of secreted PGA 2.6-fold compared to the culture without CaCl_2 _addition. Furthermore, addition of 5 mM CaCl_2 _resulted in lower amounts of biomass which is probably due to growth inhibition by higher concentration of calcium ions (data not shown). Therefore, 2.5 mM CaCl_2 _were added to the growth medium for recombinant PGA production in all following experiments.

### Characterization of secreted *B. megaterium *PGA

The *pga *gene was initially cloned with the 5' region encoding its mature signal peptide SP_*pga*_. SDS-PAGE analysis of the extracellular proteins of recombinant *B. megaterium *carrying pRBBm23 (encoding SP_*pga*_*-*PGA) revealed two subunits of PGA with relative molecular masses (M_r_) of 27,000 (α-chain) and 57,000 (β-chain) (Fig. 1). The N-terminal amino acid analysis of both recombinant exported proteins indicated that the α-chain started at amino acid residue 25 (GEDKNEGVKVVR) while the N-terminal amino acid sequence of the β-chain SNAAIVGSEKSATGN corresponded to residues 266 to 279. Hence, the α- and β-subunit of PGA range from residue 25 to 265 and from 266 to 802 with calculated molecular masses of 27,753 Da and 61,394 Da, respectively. These calculated masses corresponded well to the experimentally observed masses of the subunits and suited perfectly the report by Panbangred et al. [9]. The native signal peptide sequence was deduced as MKTKWLISVIILFVFIFPQNLVFA.

### The signal peptide of the extracellular lipase LipA increases PGA export in *B. megaterium*

In previous works, the signal peptide of the *B. megaterium *extracellular esterase LipA (SP_*lipA*_) was successfully used for the secretion of *Lactobacillus reuteri *levansucrase [6] and *T. fusca *hydrolase [8]. In order to analyze the efficiency of the LipA signal peptide for the secretion of recombinant *B. megaterium *PGA, protein secretion mediated by SP_*lipA *_and by its natural signal peptide (SP_*pga*_) were compared. *B. megaterium *strain MS941 carrying the plasmid pRBBm49 encoding a SP_*lipA*_-PGA fusion and the plasmid pRBBm23 encoding the native PGA (SP_*pga*_-PGA), respectively, were cultivated in LB medium. A maximum of 380.0 and 230.0 U PGA g_CDW _^-1 ^were measured for the exported PGA using the SP_*lipA *_and SP_*pga*_, respectively. Hence, changing the original signal peptide of PGA to the one of LipA improved the amount of secreted PGA 1.7-fold (Tab. 2).

**Table 2:** Stepwise improvement of PGA production and export using *B. megaterium*

Strain	Plasmid	Medium*	Cultivation	PGA activity [U g_CDW _^-1^]	PGA [mg L^-1^]
MS941	pRBBm23	A5	SF	6.0	0.3
MS941	pRBBm23	A5	Batch	17.0	4.2
MS941	pRBBm23	LB1	SF	230.0	25.0
MS941	pRBBm49	LB1	SF	380.0	36.0
MS941	pRBBm49	LB2	SF	500.0	20.0
YYBm1	pRBBm23	LB1	SF	280.0	33.0
YYBm1	pRBBm49	LB1	SF	390.0	41.0
YYBm1	pRBBm49	LB2	SF	830.0	22.0
YYBm1	pRBBm49	MM	SF	0.0	0.0
YYBm1	pRBBm49	MM + 0.5 × AA	SF	170.0	11.0
YYBm1	pRBBm49	MM + 1 × AA	SF	330.0	35.0
YYBm1	pRBBm49	MM + 2 × AA	SF	200.0	28.0
YYBm1	pRBBm49	LB2	Batch	640.0	25.0
YYBm1	pRBBm49	MM + 1 × AA	Batch	320.0	29.0

*all media were supplemented with 2.5 mM CaCl

LB1 includes tryptone from Oxoid

LB2 was prepared with tryptone from Bacto

AA: amino acid solution

MM: MOPSO based minimal medium

SF: shaking flask cultivation.

Batch: pH controlled fermentation.

pRBBm23: *B. megaterium pga *gene encoding its natural leader sequence.

pRBBm49: *B. megaterium pga *gene encoding the LipA leader sequence.

The purified enzyme has a specific activity of 45 U mg_protein _^-1 ^[27]. Standard derivations performed experiments were below 10 %.

### Construction of a *B. megaterium *strain deficient in the utilization of the gene expression inducing xylose

**Figure 2:**
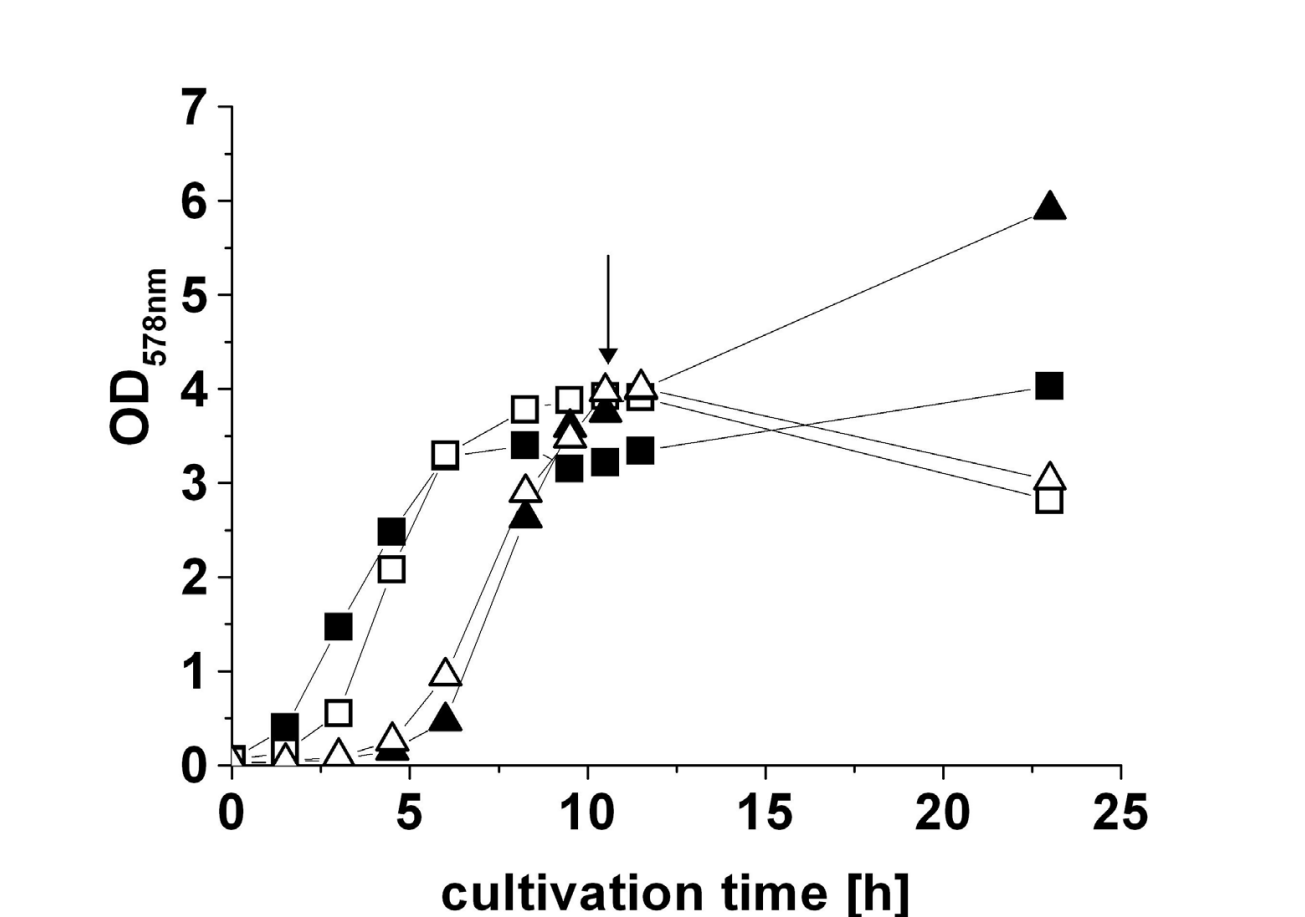
*B. megaterium *YYBm1 is deficient in xylose utilization. Shaking flask cultivation of *B. megaterium *strain MS941 (Δ*nprM*) (■), YYBm1 (Δ*nprM*, Δ*xylA*) (□), WH320 (▲), and WH323 (Δ*xylA*) (△) in minimal medium with glucose as initial carbon source. At the beginning of the stationary phase 5 g L^-1 ^xylose was added as second carbon source into the growth medium (indicated by arrow).

HPLC analysis of growth medium of batch cultivations with MS941 carrying pRBBm23 (encoding SP_*pga*_-PGA) in A5 medium indicated the utilization of xylose as carbon source after the majority of glucose in the growth medium was consumed (data not shown). In order to improve target gene induction efficiency, a constant level of the inducer xylose during cultivation had to be guaranteed. This was achieved by constructing a stable strain deficient in xylose utilization [4]. In agreement with this assumption, the use of the *xylA *knock-out mutant strain *B. megaterium *WH323 in protein production using the xylose inducible promoter resulted in higher yields of intracellularly produced heterologous protein [15]. However, a major drawback of WH323 was an increased secretion of the neutral extracellular protease NprM [8]. *B. megaterium *MS941 employed in this study lacks NprM [1,5] Hence, a strain deficient in xylose utilization based on *B. megaterium *MS941 was constructed by interrupting the gene encoding the xylose isomerase XylA with the *cat *gene mediating chloramphenicol resistance. The new strain was named YYBm1. To compare their sugar metabolization, *B. megaterium *strains MS941, WH320, YYBm1, and WH323 were cultivated in minimal medium with glucose as sole carbon source. When glucose in the growth medium was consumed, all *B. megaterium *strains stopped growing and entered the stationary phase. After addition of xylose as second carbon source, the strains MS941 and WH320 were entering a second exponential phase of growth, whereas cells of the strains YYBm1 and WH323 died. Hence, YYBm1 and WH323 were unable to utilize xylose as carbon source (Fig. 2). Consequently, the *xylA nprM *double mutant YYBm1 revealed the expected phenotype. When tested in protein production experiments, YYBm1 secreted 390.0 U PGA per gram CDW compared to 380.0 U PGA per gram CDW by MS941 (Tab. 2). Comparing the two strains for the export of PGA carrying its natural leader peptide an increase of 1.2-fold in the specific activity was observed (Tab. 2).

Next, early and late induction of gene expression by the addition of xylose were compared. When the inducer xylose was added right at the beginning of the cultivation, the maximal specific activity was reached 7.5 h after the start of cultivation. Similar final activities were reached when xylose was added at an OD_578nm _of 0.4 (data not shown). An induction of gene expression at higher optical density, *e.g*. at OD_578nm _4, led to a faster appearance of PGA activity after induction, but just half the amount of PGA was obtained compared to the early induction (data not shown). Hence, 5 g L^-1 ^xylose was added right at the beginning of the cultivation.

### Optimization of the complex growth medium

**Figure 4:**
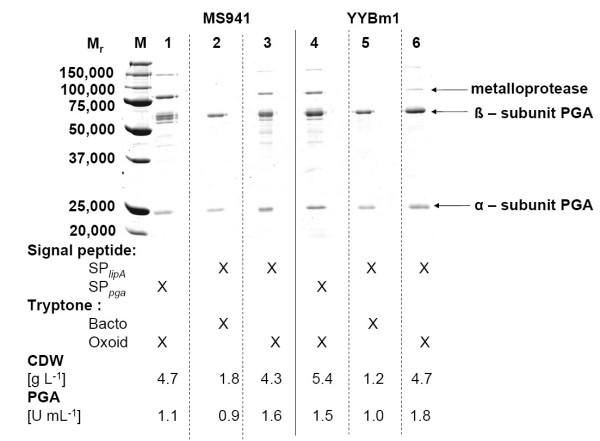
Comparison of different leader peptides for the production and export of *B. megaterium *PGA. PGA was produced in shaking flask cultivation of *B. megaterium *MS941 and YYBm1 carrying either pRBBm23 (encoding SP_*pga*_-PGA) or pRBBm49 (encoding SP_*lipA*_-PGA) in LB medium containing tryptone from different companies. At OD_578nm _of 0.4 *pga *expression was induced by the addition of 5 g L^-1 ^xylose to the growth medium. Samples were taken at various time points after induction. Proteins from 10 μL unconcentrated growth medium were separated by SDS-PAGE and stained with Coomassie Brilliant Blue G250. Biomass concentration and PGA volumetric activity 24 h after induction of recombinant gene expression are shown.

Next, the effects of the addition of tryptones from two different companies to the complex growth medium were investigated. PGA secretion by MS941 carrying pRBBm49 (encoding SP_*lipA*_-PGA) in LB medium was improved 1.8-fold to 36.0 mg L^-1 ^by utilizing tryptone from Oxoid (Wesel, Germany) instead of that from Bacto (Heidelberg, Germany) (Tab. 2). These two tryptones varied in the concentrations of contained amino acids, especially in the amount of arginine, aspartic acid and tyrosine. Used Oxoid versus Bacto tryptone contain 5.53 % to 3.03 % arginine, 7.31 % to 6.11 % aspartic acid, and 3.1 % to 1.42 % tyrosine, respectively. 1.8 times more PGA (41 mg L^-1^) was secreted by YYBm1 carrying pRBBm49 (encoding SP_*lipA*_-PGA) in LB medium utilizing Oxoid tryptone compared to Bacto tryptone (Tab. 2). For MS941 and YYBm1 a maximal OD_578nm _of 14 were reached during cultivation with Oxoid tryptone. Only OD_578nm _of 4 and 6 were reached by MS941 and YYBm1, respectively, when grown in LB containing Bacto tryptone. Interestingly, in contrast to the volumetric activity the specific activity is 1.4- and 2-fold higher for PGA obtained from cultivations of *B. megaterium *MS941 and YYBm1 using tryptone from Bacto instead of Oxoid, respectively (Tab. 2). Another difference in cultivation with these two media was the production of an extracellular immune inhibitor A metalloprotease like protein Q73BM2 (M_r _= 84,400) in the presence of tryptone from Oxoid (Fig. 4). The production of this protein was observed before for *B. megaterium *by Wang et al. (2006) [16]. The protein was identified using the MASCOT program with MALDI-TOF/MS data and the strain-specific protein database "bmgMECI".

### From complex to mineral medium

**Figure 3:**
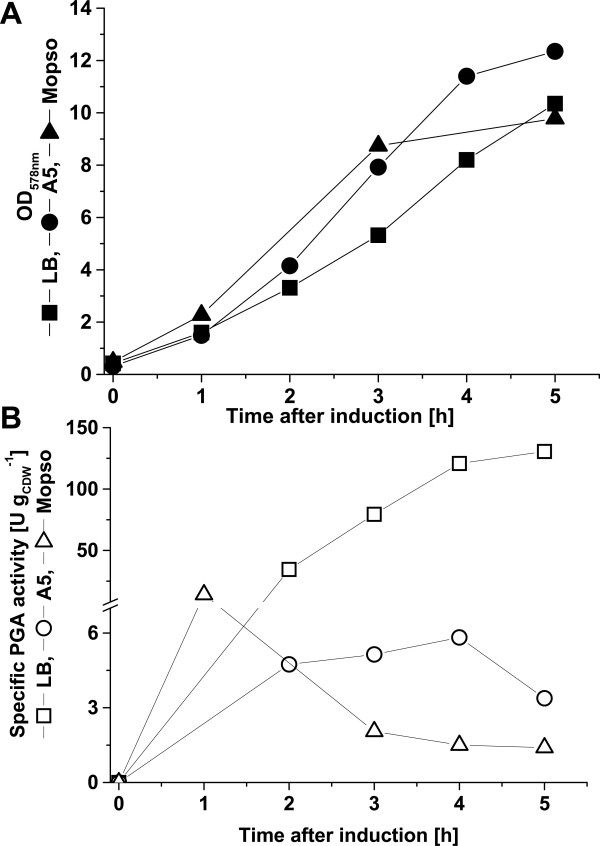
Comparison of growth media for PGA production and export using *B. megaterium*. MS941 carrying pRBBm23 (encoding SP_*pga*_-PGA) grew in LB (square), A5 (circle), and MOPSO (triangle) medium. The *pga *expression was induced at OD_578nm _of 0.4 by adding 5 g L^-1 ^xylose. (**A**) Solid symbols represent the measured growth curve. (**B**) open symbols represent specific PGA activity.

For the control and subsequent directed optimization of the fermentation process defined mineral media are desired. Moreover, these mineral media usually are less cost intensive compared to complex media. Therefore, we systematically developed a mineral medium for PGA production and export in *B. megaterium*. First, the previously developed semi-defined A5 medium [1] containing 0.5 g L^-1 ^yeast extract and a newly developed mineral medium based on MOPSO buffer were tested in comparison to complex medium. Growth and secretion of PGA were initially compared for the different media in shaking flask cultivations of *B. megaterium *MS941 carrying pRBBm23 (encoding SP_*pga*_-PGA) (Fig. 4). Using complex medium, maximal specific PGA activity of 131 U g_CDW _^-1 ^was reached 5 h after induction of *pga *expression. A cultivation in semi-defined A5 medium led to a drastic 22-fold reduction (maximum of 6.0 U g_CDW _^-1^) while in MOPSO derived medium specific PGA activity was reduced 9.4-fold (maximum of 14 U g_CDW _^-1^) (Fig. 3). Although the MOPSO derived mineral medium was a protein and amino acid free medium, similar cell densities were reached compared to complex medium. In addition, higher specific PGA activities compared to the semi-defined A5 medium were achieved. Hence, we started to optimize the MOPSO-based medium by systematic supplementation of nutrients to increase PGA production and export.

**Figure 5:**
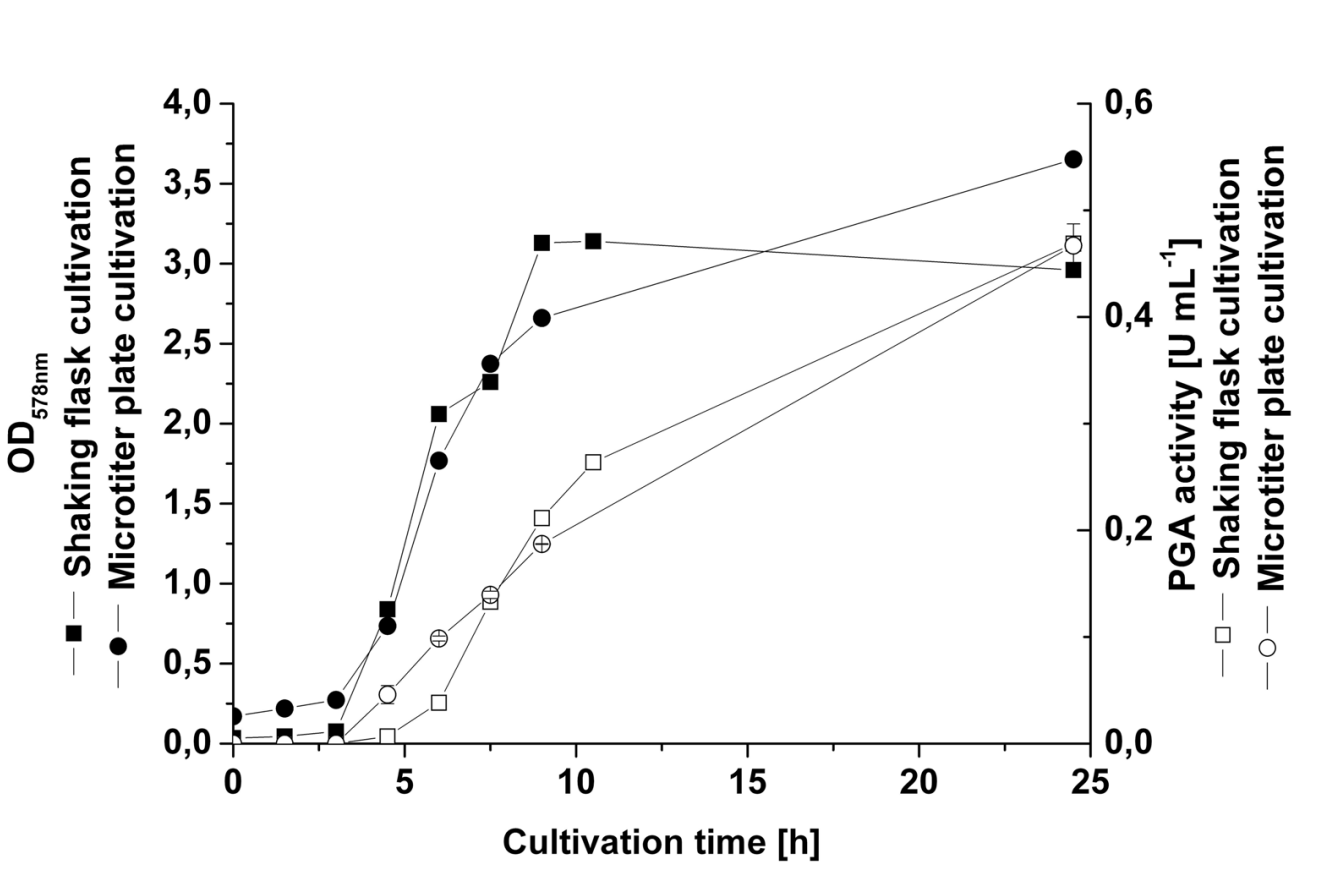
Cultivation and PGA production in microtiter plates. YYBm1 carrying pRBBm49 (encoding SP_*lipA*_-PGA) was cultivated in LB medium using microtiter plates and shaking flasks. OD_578nm _from microtiter plate cultivation was measured with a spectrophotometer and Multiskan Ascent photometer. PGA activity measurements were performed as described in material and methods.

Acevedo et al. (1973) and Pinotti et al. (2000) showed that for high production of PGA in *B. megaterium *ATCC14945 certain amino acids were required [17,18]. Hence, for improving the productivity in minimal medium, the influence of the amino acids addition on PGA secretion was investigated. Free amino acids as arginine, proline, histidine, and asparagines were selectively added to the medium including glucose as carbon source and casein as nitrogen source [18]. Growth and PGA production of *B. megaterium *YYBm1 carrying pRBBm49 (encoding SP_*lipA*_-PGA) was systematically investigated in 96-well microtiter plates. The expression of *pga *was induced at the beginning of cultivation. First, the cell growth and protein production characteristics were compared to shaking flasks cultivations using LB medium. Similar cell growth curves and comparable amounts of enzyme were achieved at the end of cultivations (Fig. 5). Hence, the microtiter plates allow a cultivation comparable to shaking flasks with the advantage of high through-put. Next, according to their corresponding metabolic pathways [19] the 20 amino acids to be added were grouped into 7 families: I. glycine and serine; II. valine, leucine and isoleucine; III. alanine; IV. glutamine, glutamic acid, proline, and arginine; V. histidine; VI. lysine, threonine, methionine, aspartic acid, cysteine, and asparagine; VII. phenylalanine, tyrosine, and tryptophan. Seven different combinations of amino acid solutions were prepared each time excluding one group of amino acids. When group II, IV or VII were excluded, specific activity of PGA increased up to 1.9-, 1.8- and 2.5-fold, respectively. The highest increase in PGA production was observed when amino acids from group VII were excluded. Group VII contains the aromatic amino acids (F, Y, W) which are usually produced from the pentose phosphate pathway. The minimal medium supplemented with all amino acids excluding group VII was chosen for the described scale-up experiments from microtiter plate over shaking flasks to the bioreactor.

**Figure 6:**
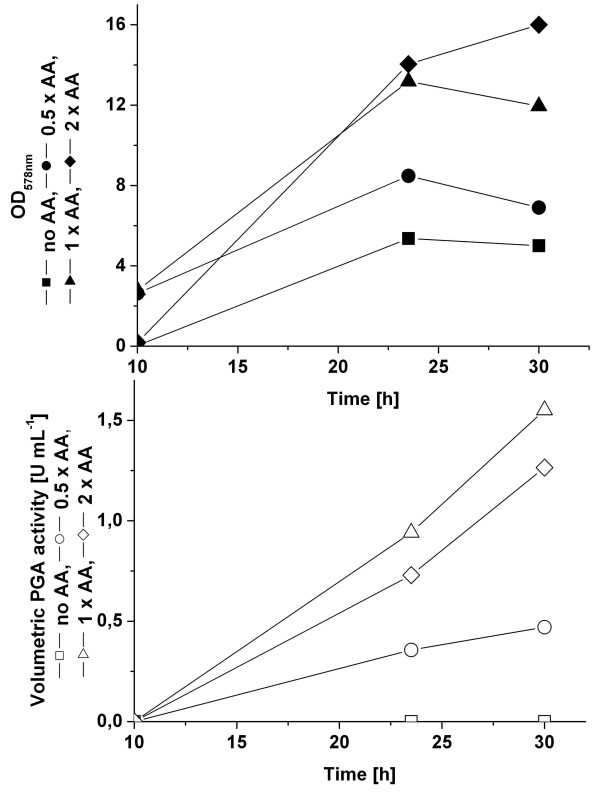
The influence of the concentration of amino acids supplementation on cell dry weight and PGA activity. Shaking flask cultivation of YYBm1 carrying pRBBm49 (encoding SP_*lipA*_-PGA) was employed.

Next, the amount of added amino acids solution was optimized in shaking flask cultivations (Fig. 6). *B. megaterium *strain YYBm1 carrying plasmid pRBBm49 (encoding SP_*lipA*_-PGA) was cultivated in 100 mL minimal medium with a final concentration of none, 0.5 ×, 1 ×, and 2 × of the amino acids solution excluding the group VII amino acids. The 2 × addition of the amino acids solution led to increased final cell density at the end of the cultivation. However, optimal PGA production was obtained in minimal medium with 1 × addition of the amino acid solution, which was also verified by SDS-PAGE analysis of extracellular proteins. These results indicated that amino acids were essential for PGA production, but the higher concentration of amino acid, here 2 ×, limited PGA production.

### Upscale of PGA production using *B. megaterium *to a 2 Liter bioreactor

**Figure 7:**
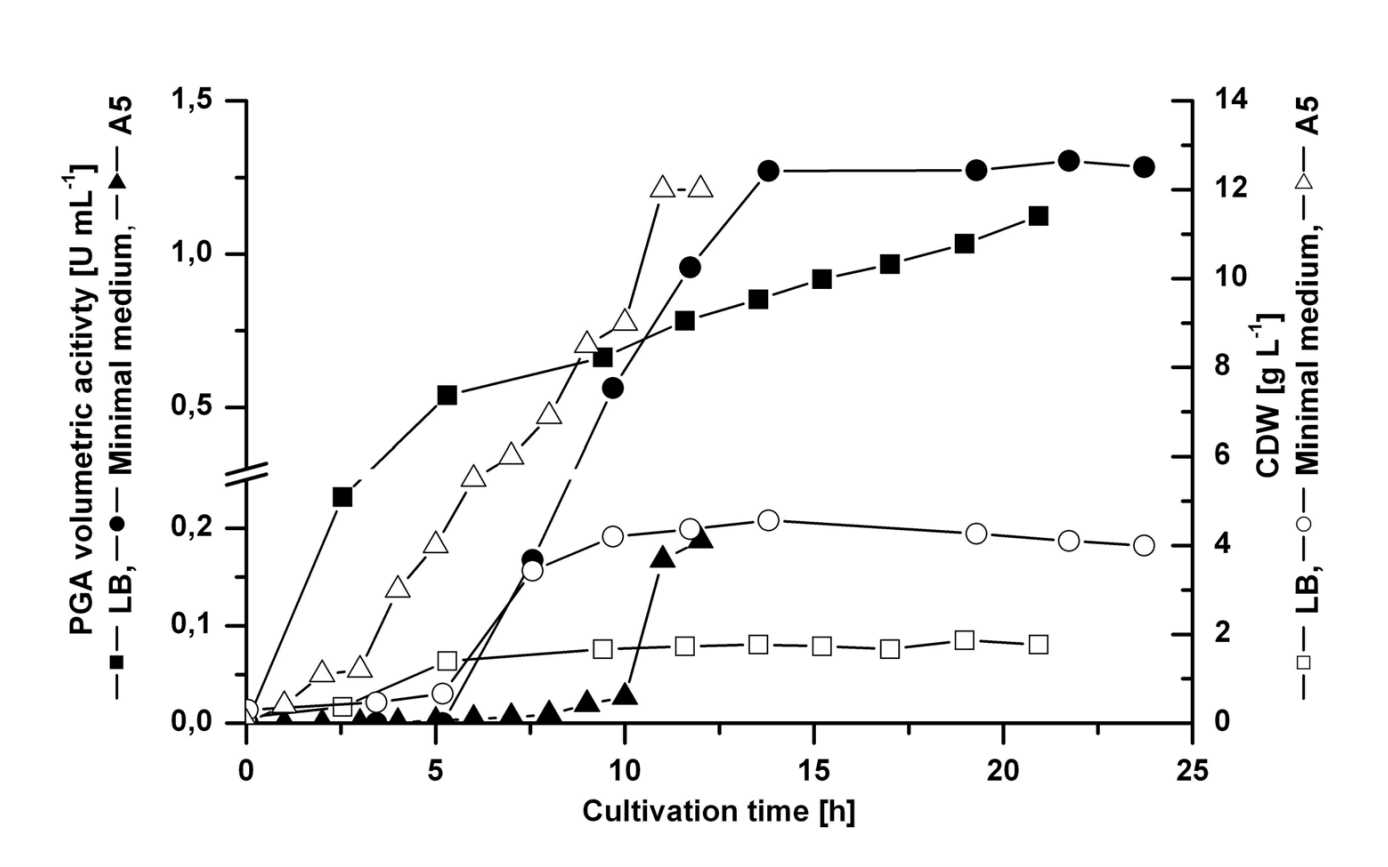
Upscaling of PGA production and export using *B. megaterium *and a 2 L bioreactor. The pH controlled batch cultivation of *B. megaterium *YYBm1 carrying pRBBm49 (encoding SP_*lipA*_-PGA) was performed in complex medium (square) and optimized minimal medium (circle). *B. megaterium *MS941 carrying pRBBm23 (encoding SP_*pga*_-PGA) was grown in semi-defined A5 medium (triangle). For induction of recombinant gene expression 5 g L^-1 ^xylose were added at the beginning of the cultivation. Samples were taken at indicated time points to determine cell dry weight (open) and PGA volumetric activity (solid).

Finally, this optimized minimal medium containing 1x amino acids solution excluding group VII amino acids was used for an upscale with a pH controlled 2 L bioreactor (Fig. 7). As control, LB complex medium with tryptone from Bacto was also tested in the bioreactor. 29.0 mg L^-1 ^PGA were produced by YYBm1 carrying pRBBm49 (encoding SP_*lipA*_-PGA) using the optimized minimal medium. This was only a slight 1.1-fold increase compared to PGA production in the complex medium. For the first time, a higher volumetric productivity was reached in a batch cultivation using a defined minimal medium compared to an undefined complex medium. However, after cultivation in LB medium the specific PGA activity was still 2 times higher than after cultivation in minimal medium due to the 2 times higher biomass production in minimal medium. No PGA precursor was observed in the medium.

Next, the obtained improvements in the bioreactor were compared to a bioreactor cultivation performed at the beginning of the study. This comparison of the described complex and minimal medium with a pH-controlled batch cultivation of *B. megaterium *strain MS941 carrying pRBBm23 (encoding SP_*pga*_-PGA) using A5 semi-defined medium excluding calcium ions (Fig. 7) provided insights into the improvement process via the different described steps. In cultivations using either LB or minimal medium, PGA secretion started in the exponential phase, whereas in a cultivation using semi-defined A5 medium it started in the stationary phase. Finally, only 4.2 mg PGA per Liter growth medium were obtained using strain MS941 carrying pRBBm23 (encoding SP_*pga*_-PGA) in A5 medium. Hence, using the newly constructed strain YYBm1 deficient in xylose utilization, the signal peptide of LipA, an optimized minimal medium supplemented with calcium ions and a defined mix of amino acids the volumetric PGA productivity was improved 7-fold resulting in 29.0 mg PGA per Liter growth medium.

## Discussion

We systematically optimized *B. megaterium *for the recombinant production of PGA. Some unexpected observations were made. A potential metalloprotease was exclusively produced by *B. megaterium *MS941 and YYBm1 cultivated in medium containing tryptone from Oxoid and not in the presence of Bacto tryptone. The Oxoid tryptone was characterized by its higher content in arginine, aspartic acid, and tyrosine. This might have provided an external stimuli of unknown nature which induced expression of the metalloprotease gene. Determination of the corresponding mRNA levels via Northern blot analysis might help to shed some light on the observed phenomena. Similarly, PGA production was also influenced by the tryptone source as well as the amino acid composition and content of the growth medium. The observed production pattern might be the result of a complex interplay of various factors influencing growth, protein production and export as well as stress responses. Usually complex media provide better growth due to the supplement of the full set of known and unknown essential growth factors. Nevertheless, the supply of C-, N-, S- sources and other growth factors in an excess often causes stress and other regulatory responses to optimize the bacterial metabolism towards the environmental stimuli. As a consequence intracellular amino acid synthesis and protein production and export might be decreased. A system biotechnology approach with the systematic high throughput determination of transcriptome, cytoplasmic proteome, secretome and especially the metabolome for the various growth and protein production conditions will finally help us to determine the exact cellular parameters involved in the observed protein production behaviour. This information might provide a solid bases for the directed further metabolomic engineering of *B. megaterium *for optimal protein production and export.

In contrast to the complex explanations for the outlined observations the more efficient PGA production and secretion via an induction of *pga *gene expression at low cell densities compared to high cell densities might simply be caused by the longer gene induction and protein production time. This phenomena was observed before by our group [8]. Once one step of protein production like protein export becomes limiting, longer protein production times increase the overall product yield. Nevertheless, produced PGA amounts (~2000 U L^-1^) in this study did not completely reach such of previously described *B. megaterium *PGA production strains (~3000 U L^-1 ^in [17], ~9000 U L^-1 ^in [9]) or that of the published intracellular production of the enzyme in *E. coli *(~30,000 U L^-1 ^in [20]). Outlined enzyme activity results are not simple to compare since absolute protein amounts are not given by the mentioned PGA productions. Therefore, observed differences between the various *B. megaterium *production strains might be due to differences in the employed enzymatic test systems. Currently, intracellular protein production in *E. coli *is still more efficient compare to recombinant protein production and export with *B. megaterium*. Limitations in up scaling protein production processes including protein export were observed for *B. megaterium *[1,6]. Again, a system biology approach should help us to identify existing bottlenecks and allow for systematic bioengineering solutions.

## Conclusion

A systematic improvement of the recombinant production and export of *B. megaterium *ATCC14945 penicillin G amidase using *B. megaterium *was performed. The addition of 2.5 mM calcium ions increased the specific activity by 2.6-fold. Exchange of its natural signal peptide by the one of the *B. megaterium *extracellular lipase LipA increased secretion by 1.7-fold. A *B. megaterium *strain deficient in the extracellular protease NprM and in xylose utilization (Δ*xylA*) was developed allowing for stable extracellular proteins and long time induction of gene expression by xylose. Next, a defined minimal medium with defined amino acid additions for high yield PGA production was developed. Finally, PGA production successfully scaled up to 2 L controlled batch fermentations.

## Materials and methods

### Plasmids and strains

All strains, plasmids and primers (biomers, Ulm, Germany) used in this study are listed in table 1. Molecular biology methods were described previously [21]. The complete wild type *pga *gene including its native signal peptide was amplified by PCR using isolated genomic DNA from *B. megaterium *ATCC14945 as template and the primers pga_23_for and pga_23_rev. After *Bsr*GI/*Sac*I digestion of the PCR product, it was cloned into the *Bsr*GI/*Sac*I site of pMM1522 [6]. The new vector was named pRBBm23. The *pga *gene was combined with the signal peptide of LipA by cloning the PCR product generated using *B. megaterium *ATCC14945 DNA and the primers pga_49_for and pga_49_rev into *Bgl*II/*Eag*I cut pMM1525 [6] generating pRBBm49.

**Table 1:** Strains, plasmids and primers used in this study.

Name	Description	Reference/source
**Strains**		
***B. megaterium***		
WH320	Mutant of DSM319,*lacZ*^-^	[4]
WH323	Mutant of WH320,*xylA1-spoVG-lacZ*	[22]
MS941	Mutant of DSM319, Δ*nprM*	[5]
YYBm1	Mutant of MS941, Δ*xylA*, Δ*nprM*	This study
***E. coli***		
DH10B	Strain for plasmid construction	Gibco Life Technologies
**Plasmids**		
pMM1520	Shuttle vector for cloning in *E. coli *(*Ap*^*r*^) and gene expression under xylose control in *B. megaterium *(*Tc*^*r*^); P_*xylA*_-MCS	[1]
pMM1522	pMM1520 derivative – vector for intracellular protein production	[6]
pMM1525	pMM1522 derivative – vector for protein secretion into the medium; P_*xylA*_-SP_*lipA*_-MCS	[6]
pRBBm23	*sp*_*pga*_*-pga *(2,476 bp) (*B. megaterium strain *ATCC14945) cloned into *Bsr*GI/*Sac*I of pMM1522; P_*xylA*_-SP_*pga*_-*pga*	This study
pRBBm48	*pga *(2,407 bp) (*B. megaterium strain *ATCC14945) without coding sequence for *sp*_*pga *_cloned into *Bgl*II/*Eag*I of pMM1525; P_*xylA*_-SP_*lipA*_-*pga *with *Sfo*I-spacer	This study
pRBBm49	pRBBm48 without *Sfo*I-spacer; P_*xylA*_-SP_*lipA*_-*pga*	This work
pHBIntE	Shuttle vector for cloning in *E. coli *(*Ap*^*r*^) and gene expression in *B. megaterium *(*Ery*^*r*^); temperature sensitive *B. megaterium ori*	[23]
pHV33	Ap^*r *^in *E. coli*, Cm^*r *^in *B. subtilis*, Cm^*r *^in *E. coli*, Tc^*r *^in *E. coli*	[24]
pYYBm4	pHBIntE derivative with *xyl*A from *B. megaterium *DSM319 genome sequence	This study
pYYBm8	pYYBm4 derivative – *xyl*A'-*cml*-'*xyl*A	This study
**Primers**		
xylA_as	ttcatgagctcttaagtgttgttcttgtgtcattcc	
xylA_s	gcaacgagctcagcagtgtatttacttgagagg	
cml_as	tgattcatatggtcgacaaaaagaaggatatggatctggagc	
cml_s	acacctctagagtcgacacaaacgaaaattggataaagtggg	
pga_23_for	tacata*tgtaca*atgaagacgaagtggctaatatca	
pga_23_rev	tatca*gagctc*atcaatagtataggctctttatgc	
pga_49_for	ttatt*agatct*t*ggcgcc*ggggaggataagaatgaagg	
pac_49_rev	tatca*cggcca*gcataaagagcctatactattgat	
cml_for	ggttatactaaaagtcgtttgttgg	
cml_rev	cgggtgataaactcaaatacagc	
xylB_rev	cctattgattcctgctaattgg	
xylR_for	cggtgcaaatctttgatattcc	
xylR_for'	cgttaagatagtcgactcc	
xylB_rev'	ccacaataacttaggaaga	
putative4_for	ccattatatattctggggcg	
ery_s	cgtcaattcctgcatgttttaagg	
ery_antis	ccaaatcggctcaggaaaag	

A xylose deficient strain was generated from *B. megaterium *MS941 by integration of the chloramphenicol resistance mediating *cat *gene into the chromosomal copy of the *xylA *gene via a double crossover [22]. For the necessary construct, the *xylA *gene was amplified by PCR from *B. megaterium *MS941 genomic DNA using the primers xylA_as and xylA_s. After digestion of the PCR product, the DNA fragment was cloned into the *Sac*I/*Sac*I sites of pHBIntE [23]. The resulting plasmid was called pYYBm4. The plasmid contained a temperature sensitive origin of replication. The *cat *gene was amplified by PCR from pHV33 [24] using the primers cml_as and cml_s and cloned into the *Nde*I/*Xba*I sites of pYYBm4. The resulting plasmid was called pYYBm8. The constructed plasmid was transformed into protoplasted *B. megaterium *MS941. Cells were grown at a non-permissive temperature of 30°C [3]. The double crossover was achieved by dividing the chromosomal integration process into two screenable step: First, single-crossover recombination was achieved by cultivation of the culture at 42°C and addition of 3 mg L^-1 ^chloramphenicol. Second, excision of the carrier replicon was screened via isolation of chloramphenicol resistant bacteria deficient in xylose utilization. The new strain *B. megaterium *YYBm1 grew on chloramphenicol M9 agar plates and exclusively used glucose as carbon source.

Constructed expression plasmids pRBBm23 and pRBBm49 were transformed in *B. megaterium *strains MS941, YYBm1, WH320, and WH323 by protoplast transformation [3]. All used strains are derivatives of the wild type strain DSM319. MS941 has a defined deletion in the gene of the major extracellular protease NprM [5]. WH323 is derived from WH320 (a chemically obtained β-galactosidase deficient mutant of DSM319) by inserting the *E. coli lacZ *gene in the *xyl*A gene. Hence, YYBm1 and WH323 do not consume xylose as carbon source.

### Growth medium composition

As **complex medium **a high salt Luria-Bertani broth (LB) medium containing of 5 g L^-1 ^NaCl, 5 g L^-1 ^yeast extract, and 10 g L^-1 ^tryptone from Bacto (Heidelberg, Germany) or Oxoid (Wesel, Germany), respectively, was used.

The **semi-defined A5 medium **contained 30 g L^-1 ^glucose, 5 g L^-1 ^(NH4)_2_SO_4_, 2.2 g L^-1 ^KH_2_PO_4_, 300 mg L^-1 ^MgSO_4 _× 7H_2_O, 500 mg L^-1 ^yeast extract, and 2 mL L^-1 ^trace element solution. The trace element solution contained 40 g MnCl_2 _× 4H_2_O, 53 g CaCl_2 _× 2H_2_O, 2.5 g FeSO_4 _× 7H_2_O, 2 g (NH_4_)_6_Mo_7_O_24 _× 4H_2_O, and 2 g CoCl_2 _× 6H_2_O per Liter.

The **minimal medium **contained 50 mM MOPSO (pH 7.0), 5 mM Tricin (pH 7.0), 520 μM MgCl_2 _× 6H_2_O, 276 μM K_2_SO_4_, 50 μM FeSO_4 _× 7H_2_O, 2.5 mM CaCl_2_, 100 μM MnCl_2 _× 4H_2_O, 50 mM NaCl, 10 mM KCl, 37.4 mM NH_4_Cl, 1.32 mM K_2_HPO_4_, 0.4 % (w/v) glucose, 1 mL L^-1 ^trace element solution, and 1 mL L^-1 ^vitamin solution with 5 g L^-1 ^xylose for induction of recombinant gene expression. The trace element solution contained 3.708 mg (NH4)_6_Mo_7_O_24 _× 4H_2_O, 24.73 mg H_3_BO_3_, 7.137 mg CoCl_2_, 2.497 mg CuSO_4_, 15.832 mg MnCl_2_, and 2.875 mg ZnSO_4 _per Liter. The vitamin solution consisted of 6 mg biotin, 20 mg niacin amid, 20 mg p-amino benzoate, 10 mg Ca-panthotenate, 100 mg pyridoxal/HCl, 20 mg folacid, 50 mg riboflavin, 50 mg DL-6,8-thioctic acid, and 10 mg thiamine dichloride per Liter. For medium optimization, minimal medium was supplemented with different concentrations of amino acid solution. Stock solution (10 x) was prepared separately according to the maximal solubility of amino acids in water as: 10 mg alanine, 10 mg arginine, 1 mg aspartic acid, 1 mg cysteine, 40 mg glycine, 4 mg isoleucine, 2 mg leucine, 10 mg lysine, 5 mg methionine, 5 mg proline, 2.5 mg phenylalanine, 5 mg serine, 5 mg threonine, 1.6 mg glutamic acid, 1 mg tryptophane, 55 μg tyrosine, 8 mg valine, 4 mg histidine, 3 mg asparagines, and 3 mg glutamine per Liter. The various optimized amino acid solutions (1 x) were added to the growth medium as indicated.

For **solid media **15 g agar per Liter was added. For selection of *Bmegaterium *deficient in xylose utilization, M9 media were used consisting of 500 mg NaCl, 1 g NH_4_Cl, 3 g KH_2_PO_4_, 7.5 g Na_2_HPO_4 _2H_2_O, 4 g glucose, 120 mg MgSO_4_, and 10 mg CaCl_2 _per Liter tap water [25].

### Cultivation

*B. megaterium *precultures were cultivated in 50 mL of the indicated medium at 37°C and 120 rpm for 16 h. For microtiter plate cultivation, 200 μL culture medium with an adjusted initial OD_578nm _of 0.1 to 0.2 was transferred to a 96-well microtiter plate except the outer wells which were filled with water because of the evaporation. The plate was cultivated in the Fluoroskan Ascent fluorescence reader (Thermo electron corporation, Dreieich, Germany) at 37 C and 1,020 rpm with an orbital shaking diameter of 1 mm as described previously [26].

For **shaking flask cultivation**, *B. megaterium *strains were grown in 500 mL baffled Erlenmeyer flasks with 100 mL medium at 37°C and 250 rpm. Expression of the *pga *gene was induced by addition of 5 g L^-1 ^xylose to the growth medium.

For **bioreactor cultivation**, a Biostat B2 (B. Braun, Melsungen, Germany) with 2 L working volume connected to an exhaust gas analysis unit (S710, Sick Maihak, Germany) was used. The bioreactor was inoculated with 1 % (v/v) cells and cultivated at 37°C with controlled pH at 7 as previously described [1,8].

### Analytical procedures

In microtiter plate cultivation OD_580nm _was measured in the Multiskan Ascent photometer (Thermo electron corporation, Dreieich, Germany). The relationship between OD_580nm _measured from microtiter plate and OD_578nm _measured from 1 cm cuvette was determined as OD_578nm _= 3.719 × OD_580nm_. For shaking flask and bioreactor cultivation samples for biomass, metabolites, and PGA activity were taken at regular intervals. The OD_578nm _was measured in triplicates with an Ultrospec 3100 pro spectrophotometer (Amersham Pharmacia, UK). The relationship between CDW and OD_578nm _was determined as CDW [g L^-1^] = 0.395 × OD_578nm _for YYBm1 and as CDW [g L^-1^] = 0.334 × OD_578nm _for MS941. The concentration of glucose and metabolites was determined by HPLC (Shimadzu, Japan) using an Aminex HPX-87H column (Biorad, USA) and 10 mM H_2_SO_4 _as the mobile phase. A flow rate of 600 μL min^-1 ^at 60°C was used in order to separate xylose from pyruvate.

SDS-PAGE was performed using a Mini Protean 3 apparatus (Bio-Rad, USA). Proteins were stained by Coomassie Brilliant Blue G250. For N-terminal sequencing, the separated proteins were transferred onto a polyvinylidene difluoride (PVDF) membrane using a Trans-Blot Semi-Dry Transfer Cell (Bio-Rad, Munich, Germany) as described by the manufacturer and the N-terminal amino acid sequence was determined by Edmann degradation.

Directly after sampling, PGA activity was measured spectrophotometrically (Ultrospec 3100 pro, Amersham Biosciences, Sweden) via release of the 6-nitro-3-phenylacetamido-benzoic acid (NIPAB) as described previously [20]. Freshly prepared NIPAB solution was prepared by dissolving 60 mg 6-nitro-3-phenylacetamido-benzoic acid in 100 mL 50 mM Na-Phosphate buffer. After addition of the enzyme sample, the absorption was immediately measured at 405 nm and 37°C for 60 s after a 20 s delay against a standard without addition of enzyme. One unit was defined as the amount of enzyme that caused the release of 1 μmol 6-nitrophenol per minute under the test conditions. The extinction coefficient of 6-nitrophenol is 8.98 cm^2 ^μmol^-1^.

## Competing interests

The author(s) declare that they have no competing interests.

## Authors' contributions

R. B and M. G carried out the molecular genetic studies, W. W participated in the sequence alignment of the metalloprotease. Y. Y participated in the design of the study and performed the statistical analysis. D. J, M. M and W. – D. D conceived of the study, and participated in its design and coordination. All authors read and approved the final manuscript.
